# Immunohistochemical Expression of PAX8 in Central Nervous System Hemangioblastomas: A Potential Diagnostic Pitfall for Neuropathologists

**DOI:** 10.1097/PAI.0000000000001246

**Published:** 2025-01-27

**Authors:** Giuseppe Broggi, Jessica Farina, Valeria Barresi, Francesco Certo, Giuseppe Maria Vincenzo Barbagallo, Gaetano Magro, Rosario Caltabiano

**Affiliations:** * Department of Medical and Surgical Sciences and Advanced Technologies “G.F. Ingrassia,” Anatomic Pathology, University of Catania; ‡ Department of Neurological Surgery, Policlinico “G. Rodolico-S. Marco” University Hospital, Catania; † Department of Diagnostics and Public Health, University of Verona, Policlinico G.B. Rossi, Verona, Italy

**Keywords:** diagnostic pitfall, differential diagnosis, immunohistochemistry, hemangioblastoma, PAX8

## Abstract

The histologic differential diagnosis between intracranial hemangioblastoma (HB) and metastatic clear cell renal cell carcinoma may be challenging, especially considering that both tumors exhibit clear cell morphology and can be associated with *vHL* mutation and/or Von Hippel-Lindau syndrome. As the execution of immunohistochemical analyses is often mandatory, the expression of PAX8 has been traditionally considered a reliable marker of metastatic clear cell renal cell carcinoma, being consistently negative in intracranial HB. However, as in recent years, some cases of PAX8-positive HBs have been reported in the literature; we studied the expression of this antibody on a series of 23 intracranial HB, showing that about 40% of these tumors may express PAX8 and that this immunoreactivity is often focal and weak. We would like to emphasize that the possibility of a PAX8-positive intracranial HB does exist and must be taken into account by neuropathologists to avoid misdiagnoses; in this regard, a broader immunohistochemical panel also including CD10, Inhibin-α, PAX2, S100, and anti-Renal cell carcinoma (RCC) antibody is highly recommended.

Hemangioblastoma (HB) is a relatively uncommon, slow-growing, central nervous system tumor of uncertain histogenesis, that exhibits a good prognosis, so that the World Health Organization assigns it a grade 1.^1,2^ Although HB most commonly involves the posterior fossa (7% to 12%), with the cerebellum being the most frequent site, it can also arise in cerebral hemispheres (1.5% to 2.5%), medulla, spinal cord and in extracranial sites.^3^ Despite the majority of HB cases are associated with sporadic mutations (loss or inactivation) of *vHL* gene (75%), it can also occur in the setting of Von Hippel-Lindau (VHL) syndrome (25%).^1–3^ Radiologically, HB more often arises as a cystic mass, but it can also show a solid or a solid-cystic growth pattern.^1–3^ Although the tumor often appears as a well circumscribed lesion without true capsule, but a focal infiltration of the surrounding brain parenchyma may be occasionally seen.^4^ Histologically, HB typically exhibits 2 main components: large neoplastic stromal cells with clear/foamy cytoplasm with lipid-containing vacuoles and abundant reactive vascular cells forming thin-walled, variable-sized vessels.^4^ Based on which of the 2 components predominates, 2 histologic variants are distinguished: (1) reticular HB (vascular cells > stromal cells) and (2) cellular HB (stromal cells > vascular cells).^4,5^ HB may sometimes show degenerative atypia, but necrosis is generally absent, and mitoses are absent to rare. Recently, DNA methylation profiling of central nervous system HBs identified 2 distinct subgroups^5^ methylation cluster 1 included HBs of mainly cerebellar location and exhibiting frequent chromosomal copy-number alterations, and methylation cluster 2 contained HBs predominantly located in noncerebellar sites and with low chromosomal copy-number alterations.^5^

Especially in cellular tumors, the differential diagnosis between HB and metastatic clear cell renal cell carcinoma (ccRCC) is quite challenging if based solely on morphology, and it can be even more complex due to the fact that ccRCC is often associated with *vHL* mutation and/or VHL syndrome. The execution of ancillary methods, such as immunohistochemistry, is often mandatory to guide the diagnosis. Classically, the immunohistochemical panel has comprised at least the following 5 antibodies: PAX8, CD10, Inhibin-α, S100, and Renal cell carcinoma (RCC).^6^ Although PAX8 has always been considered the most useful marker to solve this diagnostic problem, being the most sensitive and specific marker of renal origin of a metastatic carcinoma and consistently negative in HBs, recently, some cases of HBs have been reported as positively stained with PAX8 by some authors,^7^ suggesting that PAX8 should be used cautiously in this differential diagnostic context and preferably in association with other antibodies.

The present retrospective study aimed to evaluate the immunohistochemical expression of PAX8 on a series of 23 cases of intracranial HB, emphasizing the potential diagnostic pitfalls for the pathologist in the differential diagnosis of metastatic ccRCC.

## MATERIALS AND METHODS

The present research was in accordance with the Declaration of Helsinki and obtained the approval of the Local Ethics Committee, Catania 1 (CE 165/2015/PO). All the patients involved gave their written informed consent.

Twenty-three cases of intracranial HB were retrieved from the pathology archive of the Department of Medical, Surgical and Advanced Technologies “G.F. Ingrassia,” at the University of Catania, and from the pathology section of the Department of Diagnostics and Public Health, at the University of Verona. Clinical data were obtained from the original pathology reports. Hematoxylin and eosin–stained slides and a variable number of immunohistochemical slides were available for each case. All the hematoxylin and eosin–stained sections were reviewed by 2 pathologists (G.B. and J.F.), and the diagnoses of HB were histologically confirmed using the current well-established morphologic and immunohistochemical criteria.

Immunohistochemical analyses were performed as previously described^8^; sections cut from paraffin-embedded blocks were deparaffinized in xylene for 15 minutes, rehydrated, and treated with 3% H_2_O_2_ for 10 minutes to block endogenous peroxidase activity, followed by extensive rinsing in double-distilled water and further rinsing for 15 minutes in 0.01 M phosphate-buffered saline, pH 7.4. After the deparaffinization, the slides from each case were incubated with a standard immunohistochemical panel, including a rabbit monoclonal antibody anti-PAX8 (EP331; ready-to-use; 1.25 g/mL; Cell Marque), a rabbit monoclonal antibody anti-CD10 (SP67; ready-to-use; 4.9 g/mL; Roche), a rabbit monoclonal antibody anti-Inhibin-α (MRQ-63; ready-to-use; 2.7 g/mL; Cell Marque), a polyclonal antibody anti-S100 protein (10 g/mL; Roche), and a mouse monoclonal antibody anti-RCC (PN-15; ready-to-use; 14.4 g/mL; Cell Marque), was applied to each case; if any of these antibodies had not been tested at the time of the original diagnosis, they were added for the current study. The immunohistochemistry for CD10, Inhibin-α, and RCC was assessed as positive if cytoplasmic staining was found, while neoplastic cells were considered immunoreactive for PAX8 and S100 if brown chromogen was found at nuclear and nuclear/cytoplasmic level, respectively.

A semiquantitative optical evaluation of the immunohistochemistry, based on the intensity and the extension of staining, was performed; the former was graded into absent, weak, moderate, or strong intensity, while the latter on 4-tiered system (<1% positive cells = negative staining; 1% to 10% positive cells = focal staining; 11% to 50% positive cells = heterogeneous staining; >50% positive cells = diffuse staining).

## RESULTS

### Clinico-pathologic Features

Table 1 summarizes the clinico-pathologic features of HB from our cohort.

**TABLE 1:** Clinico-pathologic and Immunohistochemical Features of Our Series

Case	Age (y)	Sex	Anatomic site	PAX8	Extension	Intensity
1	57	Female	Cerebellum	-	0	0
2	19	Male	Cerebellum	+	Focal	Weak
3	36	Male	Cerebellum	+	Heterogeneous	Weak
4	55	Male	Cerebellum	+	Heterogeneous	Moderate
5	46	Male	Cerebellum	+	Focal	Weak
6	23	Male	Cerebellum	-	0	0
7	39	Female	Cerebellum	-	0	0
8	53	Male	Cerebellum	-	0	0
9	49	Female	Cerebellum	+	Focal	Weak
10	72	Female	Cerebellum	-	0	0
11	60	Male	Cerebellar	-	0	0
12	38	Male	Medulla oblongata	+	Focal	Weak
13	59	Female	Medulla oblongata	-	0	0
14	87	Male	Cerebellum	-	0	0
15	76	Male	Cerebellum	+	Focal	Weak
16	58	Male	Cerebellum	-	0	0
17	58	Female	Not available	-	0	0
18	69	Male	Cerebellum	+	Focal	Weak
19	29	Male	Cerebellum	-	0	0
20	55	Female	Cerebellum	-	0	0
21	65	Female	Medulla oblongata	-	0	0
22	46	Female	Cerebellum	-	0	0
23	73	Female	Cerebellum	+	Focal	Weak

Our study included 23 patients (13 males and 10 females) with a mean age of 53.13 years (median age: 55 y; age range: 19 to 87 y) affected by HBs located at the cerebellum (19 out of 23 cases; 82.6%) and medulla oblongata (3 out of 23 cases; 13%). The exact anatomic site was not available in one case (4.4%). According to the clinical data from the original pathology reports, the association with VHL syndrome was documented in 6 out of 23 cases (26.1%), whereas the remaining tumors (17 out of 23; 73.9%) were sporadic.

**FIGURE 1:**
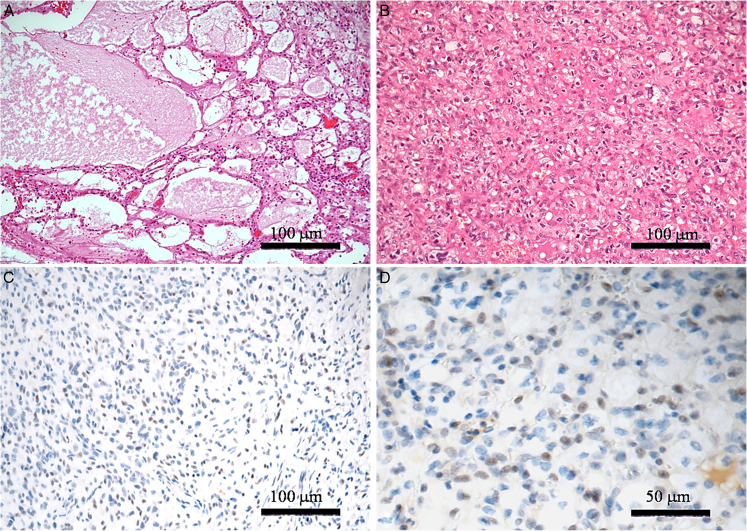
Histologic and immunohistochemical features of the reticular (A) and cellular (B) HB samples on H&E (A and B), and immunoperoxidase staining (C and D). A, Reticular subtype is predominantly composed of variable-sized capillary vessels, while neoplastic stromal cells are poorly recognizable. B, Cellular HB exhibits a striking prevalence of large stromal cells with clear/foamy cytoplasm; note the focal degenerative nuclear atypia. C and D, Neoplastic cells are weakly to moderately and focally to heterogeneously stained with PAX8 at medium (C) and high (D) magnification. Scale bars: 100 μm (A–C), 50 μm (D). HB indicates hemangioblastoma; H&E, hematoxylin and eosin.

Histologically, 17 out of 23 cases (73.9%) exhibited a prevalence of the vascular component and were classified as reticular HB subtype (Fig. 1A); 6 out of 23 cases (26.1%) showed a prevalence of stromal cells and were assigned to the cellular subtype (Fig. 1B), accordingly.

### Immunohistochemical Findings

All HBs (23 out of 23; 100%) from our cohort exhibited strong and diffuse immunoreactivity with Inhibin-α, while neoplastic cells were at least focally stained with S100 in 18 out of 23 cases (80%). Conversely, all cases were consistently negative for CD10 and RCC.

Regarding PAX immunoreactivity, it was found as positive in 9 out of 23 cases (39.1%; Fig. 1C, D), while the remaining tumors showed no expression of this protein (14 out of 23; 60.9%). Among the 9 PAX8-positive HBs, the intensity of staining was weak in almost all cases (8 out of 9; 88.9%) and moderate in one case (11.1%), whereas the extension of staining was focal in 7 out of 9 cases (77.8%) and heterogeneous in the remaining 2 tumors (22.2%).

## DISCUSSION

The immunophenotype of HB is frequently characterized by immunoreactivity for S100, Inhibin-α, and vimentin. Because of its clear cell morphology, the abundant vascular component and its association with *vHL* mutations and/or VHL syndrome, intracranial HBs must be frequently distinguished by metastatic ccRCCs; accordingly, additional immunohistochemical antibodies including PAX8, CD10 and RCC^9,10^ are often required to avoid a misdiagnosis that would be extremely deleterious for the patient as HBs are low-grade and slow-growing lesions, successfully treated with surgical resection, while surgery plus adjuvant radiotherapy are required for metastatic ccRCC. Although in the past it has been shown that metastatic ccRCC markers were all consistently negative in HB, in the last decade some authors reported that PAX8 which still represents the most sensitive marker of renal origin of a metastatic carcinoma, may be expressed by both lesions.^7^ PAX8 is one of the 9 members of the mammalian paired box family of transcription factors (PAX), and it is involved not only in crucial steps of fetal development of kidney, thyroid, and Müllerian organs, but also in neoplastic growth.^7,11^ Since PAX8 has been reported as consistently negative in all HB cases, it has always been considered one of the most reliable markers of metastatic ccRCC. Carney et al^6^ reviewed 20 HBs and 16 ccRCCs, testing all of them for PAX2, PAX8, and Inhibin-α: all 20 HBs were positively stained with Inhibin-α, PAX2 was positive in 1 case, whereas PAX8 was consistently negative in all HBs. According to these results, combined use of PAX2, PAX8, and Inhibin-α seemed to be sufficient to rule out metastatic ccRCC in the differential diagnosis with HB, with a sensitivity of 95% and a specificity of 100% in the presence of a PAX2-negative, PAX8-negative and Inhibin-α-positive tumor.^6^ Conversely, PAX8 immunoreactivity was first reported on a series of 11 cerebellar HB samples by Eichberg et al^7^: in more detail, they found an alarming positivity for PAX8 in 7 out of 11 lesions (63.6%), emphasizing that great caution must be taken by pathologists when using PAX8 alone in the differential diagnosis between HB and metastatic ccRCC. Other studies have also shown that some extra-cranial HBs did express PAX8.^12–14^ Rivera et al^15^ claimed that PAX2 is just as effective as CD10 in differentiating HB from ccRCC and did not test their cases for PAX8. In line with the latter, Carney et al^6^ emphasized that PAX2 and Inhibin-α were the best markers for this differential diagnostic purpose and that PAX8 had to be used as the second ccRCC marker, as its use increased the diagnostic sensitivity for metastatic ccRCC from 88% to 94%, without changing the specificity and the positive predictive value.^6^ Other authors reported that CD10 is a good marker for distinguishing between HB and metastatic ccRCC, being consistently negative in all HB cases.^16^

The findings of this study align with those of Eichberg et al,^7^ confirming that intracranial HBs can express PAX8. However, our larger cohort revealed a lower percentage of PAX8-positive tumors compared with the previous study (∼40% vs 60%).^7^ In addition, in most cases, PAX8 immunoreactivity was focal and weak. Thus, while the proportion of PAX8-positive HBs appears to be lower than previously reported, it is important to acknowledge that HBs exhibiting PAX8 immunoreactivity can still occur. As previously hypothesized,^7^ we also believe that the underestimation of PAX8 expression in HBs is unlikely to be due to poor reagent quality or antibody variability. Instead, it is more likely attributable to the omission of PAX8 from the standard HB immunohistochemical panel.

### CONCLUSION

Although PAX8 has always been considered a reliable marker for distinguishing HB (PAX8-negative) from metastatic ccRCC (PAX8-positive), in the present study, about 40% of intracranial HBs from our series (9 out of 23) were stained, at least focally and weakly, with this antibody. Accordingly, although we strongly emphasize that only a minority of intracranial HBs are stained with PAX8 and that this staining is often just weak and focal, neuropathologists must be aware of this unusual and unexpected staining to avoid misdiagnosis with metastatic ccRCC, especially on small biopsies. In this regard, we recommend that PAX8 is never used alone but that it is included in a broader immunohistochemistry panel, also including other markers such as CD10, Inhibin-α, PAX2, S100, and RCC.

## References

[1] Hamzah A Bamsallm M Alshammari KA . A bibliometric analysis of the top 100 cited articles for hemangioblastoma of the central nervous system. Neurosurg Rev. 2023;46:168. 37414966 10.1007/s10143-023-02070-9

[2] Kanno H Yoshizumi T Shinonaga M . Role of VHL-JAK-STAT signaling pathway in central nervous system hemangioblastoma associated with von Hippel-Lindau disease. J Neurooncol. 2020;148:29–38. 32356150 10.1007/s11060-020-03506-8

[3] Muscarella LA Bisceglia M Galliani CA . Extraneuraxial hemangioblastoma: a clinicopathologic study of 10 cases with molecular analysis of the VHL gene. Pathol Res Pract. 2018;214:1156–1165. 29941223 10.1016/j.prp.2018.05.007

[4] Yoda RA Cimino PJ . Neuropathologic features of central nervous system hemangioblastoma. J Pathol Transl Med. 2022;56:115–125. 35501672 10.4132/jptm.2022.04.13 PMC9119802

[5] Woltering N Albers A Müther M . DNA methylation profiling of central nervous system hemangioblastomas identifies two distinct subgroups. Brain Pathol. 2022;32:e13083. 35637626 10.1111/bpa.13083 PMC9616087

[6] Carney EM Banerjee P Ellis CL . PAX2(-)/PAX8(-)/inhibin A(+) immunoprofile in hemangioblastoma: a helpful combination in the differential diagnosis with metastatic clear cell renal cell carcinoma to the central nervous system. Am J Surg Pathol. 2011;35:262–267. 21263247 10.1097/PAS.0b013e3182064d11

[7] Eichberg DG Buttrick S White K . PAX8 expression variability in cerebellar hemangioblastoma: case series and review of the literature. Appl Immunohistochem Mol Morphol. 2019;27:477–481. 29629948 10.1097/PAI.0000000000000649

[8] Tirrò E Massimino M Broggi G . A custom DNA-based NGS panel for the molecular characterization of patients with diffuse gliomas: diagnostic and therapeutic applications. Front Oncol. 2022;12:861078. 35372034 10.3389/fonc.2022.861078 PMC8969903

[9] Ingold B Wild PJ Nocito A . Renal cell carcinoma marker reliably discriminates central nervous system haemangioblastoma from brain metastases of renal cell carcinoma. Histopathology. 2008;52:674–681. 18393979 10.1111/j.1365-2559.2008.03003.x

[10] Khanlou N Shintaku P Yi J . Evaluation of PAX8 expression in brain tissue and related neoplasms. Appl Immunohistochem Mol Morphol. 2016;24:207–209. 26371431 10.1097/PAI.0000000000000192

[11] Lu S Yakirevich E Hart J . PAX8 expression in breast cancer. Appl Immunohistochem Mol Morphol. 2021;29:293–298. 33208672 10.1097/PAI.0000000000000883

[12] Zhao M Williamson SR Yu J . PAX8 expression in sporadic hemangioblastoma of the kidney supports a primary renal cell lineage: implications for differential diagnosis. Hum Pathol. 2013;44:2247–2255. 23849894 10.1016/j.humpath.2013.05.007

[13] Kuroda N Agatsuma Y Tamura M . Sporadic renal hemangioblastoma with CA9, PAX2 and PAX8 expression: diagnostic pitfall in the differential diagnosis from clear cell renal cell carcinoma. Int J Clin Exp Pathol. 2015;8:2131–2138. 25973115 PMC4396212

[14] Doyle LA Fletcher CD . Peripheral hemangioblastoma: clinicopathologic characterization in a series of 22 cases. Am J Surg Pathol. 2014;38:119–127. 24145646 10.1097/PAS.0b013e3182a266c1

[15] Rivera AL Takei H Zhai J . Useful immunohistochemical markers in differentiating hemangioblastoma versus metastatic renal cell carcinoma. Neuropathology. 2010;30:580–585. 20374497 10.1111/j.1440-1789.2010.01109.x

[16] Jung SM Kuo TT . Immunoreactivity of CD10 and inhibin alpha in differentiating hemangioblastoma of central nervous system from metastatic clear cell renal cell carcinoma. Mod Pathol. 2005;18:788–794. 15578072 10.1038/modpathol.3800351

